# Supplementary material for: Meta-Analysis and Gene Set Enrichment Relative to ER Status Reveal Elevated Activity of MYC and E2F in the “Basal” Breast Cancer Subgroup
Source: PLoS One. 2009 Mar 9;4(3):e4710. doi: 10.1371/journal.pone.0004710 (PMC2650420; doi:10.1371/journal.pone.0004710)
Supplement: Dataset S1 — GSEA results from first screen. The file contains a zipped archive of the HTML results of the second GSEA screen. Reports for enriched or depleted datasets may be accessed from the “index.html” link within the my_analysis.GseaPreranked.1219975950625.rpt.zip ZIP archive. Enrichment plots are available for each of the 50 most enriched and depleted gene sets. (13.32 MB ZIP) [file pone.0004710.s001.zip › my_analysis.GseaPreranked.1219975950625.rpt.zip!//CANCER_UNDIFFERENTIATED_META_UP.html]

Details for gene set CANCER\_UNDIFFERENTIATED\_META\_UP[GSEA]

|  || Dataset | zSco.ERneg.to.ERpos\_collapsed |
| Phenotype | NoPhenotypeAvailable |
| Upregulated in class | na\_pos |
| GeneSet | CANCER\_UNDIFFERENTIATED\_META\_UP |
| Enrichment Score (ES) | 0.7030642 |
| Normalized Enrichment Score (NES) | 2.6853817 |
| Nominal p-value | 0.0 |
| FDR q-value | 0.0 |
| FWER p-Value | 0.0 |
Table: GSEA Results Summary

  

Fig 1: Enrichment plot: CANCER\_UNDIFFERENTIATED\_META\_UP      
 Profile of the Running ES Score & Positions of GeneSet Members on the Rank Ordered List

  

| PROBE | GENE SYMBOL | GENE\_TITLE | RANK IN GENE LIST | RANK METRIC SCORE | RUNNING ES | CORE ENRICHMENT || 1 | CDC20 |  |  | 9 | 7.151 | 0.0295 | Yes |
| 2 | FOXM1 |  |  | 21 | 6.760 | 0.0572 | Yes |
| 3 | CENPA |  |  | 51 | 6.315 | 0.0817 | Yes |
| 4 | CCNA2 |  |  | 52 | 6.297 | 0.1083 | Yes |
| 5 | CEBPG |  |  | 72 | 6.061 | 0.1325 | Yes |
| 6 | TRIP13 |  |  | 96 | 5.890 | 0.1556 | Yes |
| 7 | KIF2C |  |  | 124 | 5.703 | 0.1777 | Yes |
| 8 | YBX1 |  |  | 157 | 5.523 | 0.1986 | Yes |
| 9 | GGH |  |  | 161 | 5.504 | 0.2216 | Yes |
| 10 | MELK |  |  | 181 | 5.424 | 0.2431 | Yes |
| 11 | MCM6 |  |  | 198 | 5.317 | 0.2643 | Yes |
| 12 | MTHFD2 |  |  | 205 | 5.307 | 0.2863 | Yes |
| 13 | KIF14 |  |  | 221 | 5.215 | 0.3071 | Yes |
| 14 | BIRC5 |  |  | 229 | 5.191 | 0.3285 | Yes |
| 15 | SLC7A5 |  |  | 235 | 5.169 | 0.3500 | Yes |
| 16 | CKS2 |  |  | 239 | 5.145 | 0.3715 | Yes |
| 17 | GPSM2 |  |  | 291 | 4.963 | 0.3886 | Yes |
| 18 | EZH2 |  |  | 312 | 4.888 | 0.4077 | Yes |
| 19 | NCAPH |  |  | 348 | 4.799 | 0.4254 | Yes |
| 20 | RFC4 |  |  | 370 | 4.741 | 0.4438 | Yes |
| 21 | CKS1B |  |  | 416 | 4.638 | 0.4600 | Yes |
| 22 | MYBL2 |  |  | 418 | 4.629 | 0.4795 | Yes |
| 23 | MAD2L1 |  |  | 477 | 4.446 | 0.4939 | Yes |
| 24 | DLG7 |  |  | 521 | 4.306 | 0.5088 | Yes |
| 25 | CCT6A |  |  | 566 | 4.210 | 0.5233 | Yes |
| 26 | TUBB |  |  | 582 | 4.183 | 0.5398 | Yes |
| 27 | MCM2 |  |  | 630 | 4.080 | 0.5535 | Yes |
| 28 | SLC16A1 |  |  | 638 | 4.055 | 0.5701 | Yes |
| 29 | MCM3 |  |  | 656 | 4.019 | 0.5858 | Yes |
| 30 | TAP1 |  |  | 674 | 3.984 | 0.6013 | Yes |
| 31 | NCAPD2 |  |  | 727 | 3.884 | 0.6138 | Yes |
| 32 | PRDX4 |  |  | 761 | 3.821 | 0.6274 | Yes |
| 33 | H2AFX |  |  | 930 | 3.591 | 0.6299 | Yes |
| 34 | UBE2C |  |  | 994 | 3.489 | 0.6399 | Yes |
| 35 | KIF23 |  |  | 1030 | 3.442 | 0.6518 | Yes |
| 36 | PSMD14 |  |  | 1069 | 3.392 | 0.6633 | Yes |
| 37 | CDC2 |  |  | 1259 | 3.182 | 0.6624 | Yes |
| 38 | TMSB10 |  |  | 1333 | 3.100 | 0.6700 | Yes |
| 39 | PCNA |  |  | 1346 | 3.087 | 0.6821 | Yes |
| 40 | SSBP1 |  |  | 1377 | 3.060 | 0.6928 | Yes |
| 41 | CCNB1 |  |  | 1626 | 2.823 | 0.6860 | Yes |
| 42 | ILF2 |  |  | 1644 | 2.807 | 0.6966 | Yes |
| 43 | CDKN3 |  |  | 1827 | 2.674 | 0.6942 | Yes |
| 44 | NUDT1 |  |  | 1996 | 2.533 | 0.6922 | Yes |
| 45 | H2AFZ |  |  | 2105 | 2.447 | 0.6944 | Yes |
| 46 | EIF2S2 |  |  | 2127 | 2.437 | 0.7031 | Yes |
| 47 | GCLM |  |  | 2267 | 2.339 | 0.7025 | No |
| 48 | PSMD2 |  |  | 2759 | 2.063 | 0.6741 | No |
| 49 | ADRM1 |  |  | 2792 | 2.043 | 0.6803 | No |
| 50 | CDC6 |  |  | 3185 | 1.847 | 0.6586 | No |
| 51 | PSMB7 |  |  | 3335 | 1.778 | 0.6548 | No |
| 52 | CTSL |  |  | 3453 | 1.729 | 0.6533 | No |
| 53 | IFI30 |  |  | 3555 | 1.684 | 0.6528 | No |
| 54 | RAD21 |  |  | 3642 | 1.642 | 0.6532 | No |
| 55 | TOP2A |  |  | 3735 | 1.610 | 0.6531 | No |
| 56 | HMGB2 |  |  | 3883 | 1.558 | 0.6486 | No |
| 57 | POLR2K |  |  | 4289 | 1.397 | 0.6239 | No |
| 58 | KIAA0101 |  |  | 4770 | 1.230 | 0.5929 | No |
| 59 | GARS |  |  | 4828 | 1.211 | 0.5937 | No |
| 60 | CXCL9 |  |  | 5115 | 1.111 | 0.5768 | No |
| 61 | TUBB4 |  |  | 5352 | 1.025 | 0.5634 | No |
| 62 | DPM1 |  |  | 5904 | 0.847 | 0.5254 | No |
| 63 | SEC61B |  |  | 6746 | 0.575 | 0.4643 | No |
| 64 | NME1 |  |  | 7807 | 0.187 | 0.3852 | No |
| 65 | RPA3 |  |  | 11357 | -1.892 | 0.1254 | No |
| 66 | GAS6 |  |  | 12028 | -2.598 | 0.0858 | No |
| 67 | COL1A2 |  |  | 12131 | -2.748 | 0.0897 | No |
Table: GSEA details [plain text format]

  

Fig 2: CANCER\_UNDIFFERENTIATED\_META\_UP: Random ES distribution      
 Gene set null distribution of ES for **CANCER\_UNDIFFERENTIATED\_META\_UP**
